# The Efficacy of Paroxetine and Placebo in Treating Anxiety and Depression: A Meta-Analysis of Change on the Hamilton Rating Scales

**DOI:** 10.1371/journal.pone.0106337

**Published:** 2014-08-27

**Authors:** Michael A. Sugarman, Amy M. Loree, Boris B. Baltes, Emily R. Grekin, Irving Kirsch

**Affiliations:** 1 Wayne State University, Department of Psychology, Detroit, Michigan, United States of America; 2 Harvard Medical School, Boston, Massachusetts, United States of America; McGill University, Canada

## Abstract

**Background:**

Previous meta-analyses of published and unpublished trials indicate that antidepressants provide modest benefits compared to placebo in the treatment of depression; some have argued that these benefits are not clinically significant. However, these meta-analyses were based only on trials submitted for the initial FDA approval of the medication and were limited to those aimed at treating depression. Here, for the first time, we assess the efficacy of a selective serotonin reuptake inhibitor (SSRI) in the treatment of both anxiety and depression, using a complete data set of all published and unpublished trials sponsored by the manufacturer.

**Methods and Findings:**

GlaxoSmithKline has been required to post the results for all sponsored clinical trials online, providing an opportunity to assess the efficacy of an SSRI (paroxetine) with a complete data set of all trials conducted. We examined the data from all placebo-controlled, double-blind trials of paroxetine that included change scores on the Hamilton Rating Scale for Anxiety (HRSA) and/or the Hamilton Rating Scale for Depression (HRSD). For the treatment of anxiety (*k* = 12), the efficacy difference between paroxetine and placebo was modest (*d* = 0.27), and independent of baseline severity of anxiety. Overall change in placebo-treated individuals replicated 79% of the magnitude of paroxetine response. Efficacy was superior for the treatment of panic disorder (*d* = 0.36) than for generalized anxiety disorder (*d* = 0.20). Published trials showed significantly larger drug-placebo differences than unpublished trials (*d*’s = 0.32 and 0.17, respectively). In depression trials (*k* = 27), the benefit of paroxetine over placebo was consistent with previous meta-analyses of antidepressant efficacy (*d* = 0.32).

**Conclusions:**

The available empirical evidence indicates that paroxetine provides only a modest advantage over placebo in treatment of anxiety and depression. Treatment implications are discussed.

## Introduction

Antidepressant medications are prescribed to 8.7% of the US population, making them the third most common class of prescription medications [1]. Antidepressants are approved for the treatment of depression and several other mental disorders, including generalized anxiety disorder [2], panic disorder, social anxiety disorder, obsessive-compulsive disorder, and post-traumatic stress disorder [3]. While several meta-analytic investigations have been conducted examining the efficacy of antidepressants in the treatment of depression, fewer analyses have focused on the efficacy of these drugs in the treatment of other conditions, including anxiety disorders. Moreover, most meta-analyses are conducted only using published studies. However, approximately 40% of the antidepressant trials conducted by pharmaceutical companies are not published [4], [5]. Therefore, meta-analyses of antidepressant trials are prone to overestimations of effectiveness due to publication bias.

One strategy for avoiding publication bias is to conduct meta-analyses on data submitted to the Food and Drug Administration (FDA) in the process of obtaining drug approval, as the FDA requires that pharmaceutical companies provide information on all of the trials that they have sponsored [6]. However, analyses of data submitted to the FDA [5], [7]–[9] only include trials conducted prior to approval of the medications. Pharmaceutical companies often conduct additional placebo-controlled double-blind trials after the medications have been approved. Thus, the data submitted to the FDA do not represent the most complete datasets of studies conducted with the medications.

The current study addresses these potential biases by evaluating the efficacy of paroxetine, a selective serotonin reuptake inhibitor (SSRI), across all placebo-controlled double-blind studies conducted by its manufacturer, GlaxoSmithKline, including those conducted following FDA approval. As part of a 2004 lawsuit settlement, GlaxoSmithKline has been required to post online the results of all clinical trials involving its drugs on its Clinical Trial Register [10], [11]. Thus, unlike most other antidepressants, all studies of paroxetine can be evaluated without fear of publication bias. A recent meta-analysis reported that paroxetine did not significantly differ in overall efficacy from citalopram, escitalopram, fluoxetine, or sertraline in the treatment of depression [12]. Therefore, findings concerning the efficacy of paroxetine in the treatment of anxiety disorders could possibly generalize to other SSRIs, although further research would be necessary to support that proposition.

The current analysis is the first to evaluate the efficacy of an SSRI in the treatment of anxiety disorders using a complete dataset of sponsored placebo-controlled trials. Paroxetine and other SSRIs have been approved for the treatment of a variety of anxiety disorders, including generalized anxiety disorder, panic disorder, and social anxiety disorder [13]–[24]. To date, however, only two meta-analyses have investigated the degree to which SSRIs reduce symptoms of anxiety, and both of these meta-analyses focused exclusively on panic disorder [25], [26]. One of these studies [25] found a moderate advantage for antidepressants compared to placebo (*Hedge’s g* = 0.41), and the other study [26] suggested that antidepressants provide a somewhat larger benefit (Mean Effect Size = 0.55). Notably, no meta-analyses have examined anxiety disorders other than panic disorder and none have examined whether SSRIs are differentially effective in treating different types of anxiety disorders. Further, both of these meta-analyses [25], [26] observed evidence for publication bias in their analyses and did not have access to a full database of published and unpublished trials, indicating that these figures may be an overestimate of the true effect sizes. The availability of the GlaxoSmithKline Clinical Trial Register provides an opportunity to evaluate the efficacy of an SSRI in the treatment of anxiety disorders without a concern for publication bias.

The availability of a complete dataset of pre-marketing and post-marketing trials also allows for the further examination of antidepressant efficacy in the treatment of depression. Previous meta-analyses of antidepressant data obtained from the FDA have consistently revealed modest differences between drug and placebo, with mean effect sizes ranging from *d = *0.31 to 0.32 [5], [7], and raw score differences in improvement on the Hamilton Rating Scale for Depression (HRSD) [27] ranging from 1.80 to 2.51 points [7], [28]. The overall magnitude of the change in placebo-treated individuals duplicated greater than 80% of the antidepressant response [7]. The current study further evaluates the magnitude of benefit between an SSRI medication and placebo in the treatment of depression using the database of trials available through the GlaxoSmithKline Clinical Trial Register.

The goals of the current study are two-fold: 1) to determine the magnitude of benefit for paroxetine compared to placebo in the treatment of anxiety, and 2) to determine the magnitude of benefit for paroxetine compared to placebo in the treatment of depression, utilizing access to a complete database of clinical trials sponsored by the manufacturer. Studies examining antidepressant efficacy in the treatment of anxiety disorders have used a wide range of outcome measures. However, a commonly used measure across double-blind trials of anxiety disorders including generalized anxiety disorder and panic disorder is the Hamilton Rating Scale for Anxiety (HRSA) [29]. Therefore, the current study will focus on the HRSA as an indicator of anxiety-related outcomes. For both HRSA and HRSD analyses, we will analyze available moderator variables to determine which trial variables influence effect sizes in drug and placebo groups.

## Methods

### Study Retrieval

Data for all trials were obtained through the GlaxoSmithKline Clinical Trial Register [30]. According to the terms of the 2004 lawsuit, this database is required to contain every trial sponsored by GlaxoSmithKline on their medications, including paroxetine. Thus, we do not have concerns of publication bias or selective access to studies. The “result summary” files were downloaded from the website in March 2013. A total of 371 result summaries of studies on paroxetine were downloaded. Each study was evaluated for appropriateness in the current analyses. Trials were included in the current study if they met the following criteria: 1) they were a double-blind randomized intervention study containing a placebo group and at least one group receiving paroxetine; 2) they were conducted within an indicated clinical population with DSM-III or DSM-IV (depending on when the study was conducted) diagnoses of mood and/or anxiety disorders and not on healthy volunteers; 3) they included change on the HRSA and/or the HRSD from pre-treatment to post-treatment amongst their outcome measures; 4) the outcome indices were appropriately matched to the clinical diagnosis (i.e., the HRSA was evaluated in individuals with diagnoses of anxiety disorders and the HRSD was evaluated in individuals with depression); and 5) they did not include individuals who had systematically received additional treatment prior to the randomization to placebo/paroxetine. Examples meeting this last exclusion criterion include trials in which all participants were previously stabilized on another treatment and trials in which all participants simultaneously received treatment in addition to paroxetine.

Additionally, we obtained information regarding the initial approval of paroxetine from the FDA in accordance with the Freedom of Information Act [31]. This initial submission included 16 trials examining the efficacy of paroxetine in the treatment of depression and utilized the HRSD as an outcome measure. These trials have been included in previous meta-analyses of antidepressant data submitted to the FDA [7], [8]. We matched these 16 trials to their respective result summary file obtained through the GSK Clinical Trial Register. However, we observed discrepancies in sample sizes for 11 of the 16 studies (ranging from *n* = 1 to *n* = 12 for each group) between the data obtained the FDA and data from the GSK Clinical Trial Register result summaries. In all of these cases, samples were larger in the FDA datasets than in those obtained from the GSK Clinical Trial Register. In the interests of using the most complete datasets and presenting results consistent with previous meta-analyses including these trials, we used the data obtained from the FDA for these 11 trials in our analyses. Further examination revealed that the differences in sample sizes in these trials did not contribute to substantial differences in trial outcome. The overall weighted meta-analytic pre-post effect sizes for both paroxetine and placebo-treated individuals across all trials were essentially identical (within *d* = 0.002) when comparing the two data sources.

### Meta-Analytic Data Synthesis

For each outcome index (HRSA and HRSD), we conducted two types of data analysis: 1) an analysis of each trial’s arithmetic means for both groups to determine the overall meta-analytic “effect size” [32] as a comparison between the two groups (i.e., the effect size difference between paroxetine and placebo), and 2) each group’s change was calculated as the standardized mean difference, dividing the change score by the standard deviation of the change [33]. For trials that included multiple paroxetine groups compared to placebo (e.g., comparing different dosage levels or trials comparing controlled and immediate release tablets), the initial severity and change scores were combined across groups, weighted by the respective sample sizes. All analyses were conducted using the Comprehensive Meta Analysis 2.0 software package (Version 2.2.050, BIOSTAT, Englewood, NJ, USA). All analyses were conducted using both random- and fixed-effects models. Equivalent results (with regard to statistical significance) were observed for both models in almost all analyses; thus, the fixed-effects results are presented here. However, we have made the results of the random-effects models available online for interested readers (see Results S1 and Figures S1–S3). The *Q* [34] and *I^2^* [35], [36] indices were used to determine the presence or absence of homogeneity and to assess the degree of inconsistency between trials.

Analysis 1 evaluated the effect size magnitude when comparing paroxetine and placebo groups in each trial, determining the benefit of paroxetine over placebo. The effect size was calculated as the difference in the change score between groups divided by the pooled standard deviation. Analysis 2 determined the absolute magnitude of change in both the placebo and paroxetine groups for each trial (i.e., the analyses were conducted separately for each group). This latter analysis allows us to evaluate and compare the magnitude of change for both treatment conditions. For both analyses, the results are presented both in raw metric (as the mean change on the respective Hamilton rating scale) and as a standardized pre-post mean difference (*d*). The standardized mean difference results account for variation between trials in the standard deviation of the change score [37]. Weights were determined by the sample size times the inverse of the change score variance. Note that in Analysis 1 the meta-analytic weights for each study are determined by the pooled sample size and variance across both paroxetine and placebo groups, and the weights for Analysis 2 are determined for each group separately. Thus, the overall effect sizes for Analysis 1 are slightly different than the results obtained from simply subtracting the placebo from paroxetine effect sizes in Analysis 2.

We examined several moderator variables in both analyses to determine if study characteristics influenced the standardized mean difference within each treatment and/or in the comparison between paroxetine and placebo. For the HRSA, we analyzed the following moderators: 1) Baseline severity of anxiety, as determined by the mean HRSA group score at the beginning of the trial. No previous work has examined whether antidepressant and/or placebo efficacy is superior in more severe cases of anxiety, which might be predicted based on regression to the mean effects. 2) Indication (i.e., whether the individuals in the trial were treated for panic disorder or for generalized anxiety disorder). These analyses were designed to determine if the relative efficacy of paroxetine in the treatment of symptoms of anxiety varied systematically by diagnosis. 3) Length of treatment in weeks. The double-blind trials in these analyses ranged from 8 to 12 weeks; it is possible that longer trials are associated with a larger drug-placebo difference because the drug has more time to exert its effects in longer trials. Although previous studies [7], [38] have not found a significant relationship between duration of treatment and antidepressant efficacy in the treatment of depression, no previous analyses have examined this moderator variable for antidepressant efficacy in the treatment of anxiety. 4) Publication status. The current database contains all trials conducted with paroxetine, both published and unpublished; thus, publication bias is not a concern in our outcomes. Previous work [5] has demonstrated that the published literature may represent an overestimate of antidepressant efficacy in the treatment of depression, and the current analysis aimed to determine the magnitude of publication bias in the treatment of anxiety.

For the HRSD, we analyzed the following moderators: 1) Baseline severity of depression, as determined by the mean HRSD group score at the beginning of each trial. Previous analyses [7], [39], [40] have demonstrated that antidepressant-placebo differences increase with more severe depression. 2) Approval status (i.e., trials submitted to the FDA for the initial approval versus trials conducted post-approval). The 11 trials conducted following FDA approval have not been previously included in meta-analytic investigations. 3) Length of treatment in weeks. 4) Publication status.

## Results

### Study Selection

**Figure 1:**
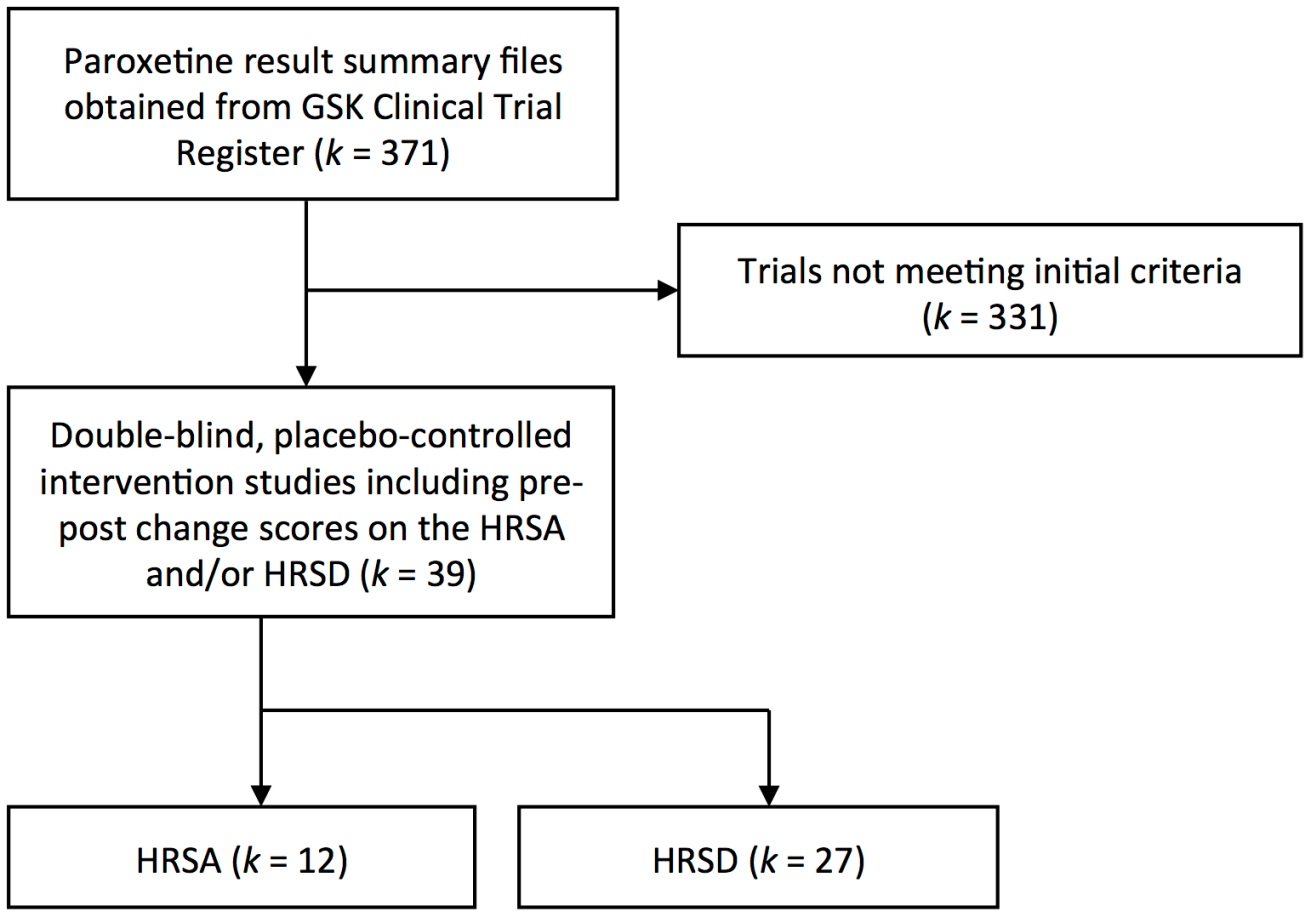
Trial selection Flow chart. GSK = GlaxoSmithKline, HRSA = Hamilton Rating Scale for Anxiety, HRSD = Hamilton Rating Scale for Depression. Please refer to the text for more specific information regarding specific exclusion criteria.

A total of 39 trials out of the original sample of 371 studies met inclusion criteria for the current analyses. The trial flow is illustrated in Figure 1. Out of the excluded studies, 121 studies did not evaluate efficacy of the drug (e.g., they evaluated the pharmacokinetics or tolerability of the drug); 153 studies were intervention studies that did not include a placebo group (e.g., they compared multiple doses of the drug, compared paroxetine against other drugs, or were open-label); 28 studies were placebo-controlled intervention studies but did not include the HRSA or HRSD in their outcome measures; 13 trials were extension studies of other trials or evaluated the efficacy of paroxetine for prevention of relapse. In nine studies, paroxetine was not the only treatment included in the intent-to-treat samples (e.g., all participants were previously stabilized on another treatment or received another simultaneous treatment in addition to paroxetine or placebo). Three studies (29060/785, 29060/251, and 29060/874) included change scores for the HRSA but the patients had a primary indication of depression rather than for anxiety disorders and thus these studies were not included. However, two of these studies (29060/251 and 29060/874) included the HRSD as an outcome measure and were included in depression analyses. Four studies included change scores on the HRSD, but the trials were for individuals with obsessive-compulsive disorder (29060/116 and 29060/118) and social phobia (29060/661 and PIR104776). The participants in these studies had low baseline severity scores (mean HRSD scores ranging from 9 to 10) and did not appear to be clinically depressed; thus, these studies were excluded. One study (29060/442) met all criteria but did not include mean change scores on the HRSD and only provided the percentage of “responders” (reduction by ≥50% on the HRSD from pre-treatment to post-treatment) in each group. Thus, we were unable to include this study in the meta-analysis.

Twelve studies were included for the HRSA, comprising 1,835 individuals randomized to paroxetine and 1,550 randomized to placebo. Twenty-seven studies were included for the HRSD, comprising 3,301 individuals randomized to paroxetine and 1,885 randomized to placebo. All studies reported their outcome measures based on “last observation carried forward” methods, meaning that the change scores for individuals who withdrew from the study were calculated based on their final data point. This method helps to control for selective attrition during the studies.

### Study Characteristics

Information on all trials is presented in Table 1. The corresponding publication information is provided where applicable. All dosage levels were within the FDA-approved range for the diagnosis. For the 12 trials evaluating change on the HRSA, trial duration ranged between 8 and 12 weeks. Five trials were 8 weeks in duration, five were 10 weeks, and two were 12 weeks. Trials were initiated between 1991 and 2003, all following FDA approval of the medication in the treatment of depression. All trials were conducted in adults. Seven trials evaluated panic disorder and five trials evaluated generalized anxiety disorder. Flexible dose adjustment was permitted in 9 of the 12 studies (i.e., the dose of paroxetine and/or placebo could be adjusted during the trial based on therapeutic response). Eight (67%) of the studies were published in peer-reviewed journals.

**Table 1:** List of studies.

Protocol Number	Publication	Outcome	Year	Indication	Population	Length	Dosage
29060/108	[18]	HRSA	1991	Panic	Adult	12	Flexible
29060/120	[14]	HRSA	1992	Panic	Adult	10	Fixed
29060/187	[16]	HRSA	1991	Panic	Adult	12	Flexible
29060/223		HRSA	1992	Panic	Adult	10	Flexible
29060/494	[21]	HRSA	1996	Panic	Adult	10	Flexible
29060/495	[21]	HRSA	1996	Panic	Adult	10	Flexible
29060/497	[21]	HRSA	1996	Panic	Adult	10	Flexible
29060/637		HRSA	1998	GAD	Adult	8	Flexible
29060/641	[20]	HRSA	1998	GAD	Adult	8	Fixed
29060/642	[19]	HRSA	1999	GAD	Adult	8	Flexible
29060/791		HRSA	2001	GAD	Adult	8	Flexible
29060A/856		HRSA	2003	GAD	Adult	8	Fixed
29060/01/001		HRSD	1984	Depression	Adult	6	Flexible
29060/02/001	[67], [68]	HRSD	1985	Depression	Adult	6	Flexible
29060/02/002	[69], [70]	HRSD	1985	Depression	Adult	6	Flexible
29060/02/003	[71]	HRSD	1985	Depression	Adult	6	Flexible
29060/02/004	[72]	HRSD	1985	Depression	Adult	6	Flexible
29060/03/001	[73], [74]	HRSD	1985	Depression	Adult	6	Flexible
29060/03/002	[75], [76]	HRSD	1985	Depression	Adult	6	Flexible
29060/03/003		HRSD	1985	Depression	Adult	6	Flexible
29060/03/004	[77]	HRSD	1985	Depression	Adult	6	Flexible
29060/03/005	[78]	HRSD	1985	Depression	Adult	6	Flexible
29060/03/006	[79]	HRSD	1985	Depression	Adult	6	Flexible
PAR 09	[80]	HRSD	1985	Depression	Adult	6	Fixed
UK 06	[81]	HRSD	1983	Depression	Adult	4	Fixed
UK 12		HRSD	1983	Depression	Adult	6	Fixed
UK 09		HRSD	1982	Depression	Adult	6	Fixed
PAR 07		HRSD	1986	Depression	Adult	6	Flexible
29060/115		HRSD	1991	Depression	Adult	12	Flexible
29060/128		HRSD	1991	Depression	Adult	12	Flexible
29060/251		HRSD	1992	Depression	Adult	8	Flexible
29060/327		HRSD	1994	Dysthymia	Adult	12	Flexible
29060/329	[82]	HRSD	1994	Depression	Adolescent	8	Flexible
29060/448	[83], [84]	HRSD	1996	Depression	Adult	12	Flexible
29060/449	[83], [84]	HRSD	1996	Depression	Adult	12	Flexible
29060/487	[85]	HRSD	1996	Depression	Elderly	12	Flexible
29060/810	[86]	HRSD	2001	Depression	Adult	8	Fixed
29060/874		HRSD	2003	Depression	Elderly	10	Fixed
112810		HRSD	2009	Depression	Adult	8	Flexible

HRSA = Hamilton Rating Scale for Anxiety. HRSD = Hamilton Rating Scale for Depression. Year: when data collection was initiated. Trials conducted prior to 1991 were submitted for the original FDA approval of the medication. Length: study duration in weeks. GAD = generalized anxiety disorder.

For the 27 trials that included change on the HRSD as an outcome measure, trial duration ranged between 4 and 12 weeks. One trial was 4 weeks in duration, fifteen were 6 weeks, four were 8 weeks, one was 10 weeks, and six were 12 weeks. Twenty-four trials evaluated change in adults, one trial evaluated change in adolescents, and two trials evaluated change in the elderly. Twenty-six trials evaluated major depressive disorder and one trial evaluated dysthymia. Flexible dose adjustment was permitted in 21 of the 27 trials. Trials were conducted between 1982 and 2009. The trials conducted prior to 1991 (*k* = 16, 59% of trials) were included as part of the original FDA submission, and an additional 11 trials (41% of trials) were conducted following FDA approval, in 1991 or later. Sixteen (59%) of the studies were published in peer-reviewed journals.

### Mean Change on the HRSA

Table 2 displays mean baseline severity, mean change, and the standardized mean difference (*d*) for each of the 12 trials reporting change on the HRSA. Baseline HRSA data were unavailable for two trials. Baseline severity of anxiety ranged from 18.7 to 26.0. The mean drug-placebo difference was 2.31 (95% CI: 1.72,2.91) points on the HRSA with a mean effect size difference of *d* = 0.27 (95% CI: 0.20,0.33). The weighted mean change on the HRSA was 11.11 (95% CI: 10.72,11.50) points for paroxetine and 8.77 (95% 8.35,9.20) points for placebo. The mean pre-post effect size was *d* = 1.23 (95% CI: 1.17,1.30) for paroxetine and *d* = 0.96 (95% CI: 0.90,1.02) for placebo. The differences between groups easily met statistical significance for both the raw change scores on the HRSA (*Z* = 7.64, *p*<.001) and the standardized mean difference (*Z* = 7.52, *p*<.001). The change in the placebo group duplicated 79% of the mean change score and 78% of the standardized mean difference in the paroxetine groups. These percentages are similar to those found for second-generation antidepressants in the treatment of depression [7].

**Table 2:** Baseline scores, mean change, standardized mean change, and sample sizes for all studies reporting change on the Hamilton Rating Scale for Anxiety.

Protocol	Indication	Paroxetine					Placebo					Drug benefit	
		Baseline	Change	*d*	95% CI	*n*	Baseline	Change	*d*	95% CI	*n*	*d*	95% CI
29060/108	Panic	24.3	14.9	2.04	[1.57, 2.51]	58	23.5	10.0	1.62	[1.21, 2.03]	56	0.74	[0.36, 1.12]
29060/120	Panic	18.9	8.4	0.95	[0.77, 1.13]	175	19.7	6.5	0.73	[0.44, 1.02]	60	0.21	[−0.08, 0.50]
29060/187	Panic		10.1	1.10	[0.87, 1.33]	119		5.9	0.59	[0.40, 0.79]	122	0.44	[0.19, 0.70]
29060/223	Panic	20.3	10.2	1.29	[0.94, 1.64]	61	18.7	5.6	0.76	[0.48, 1.04]	65	0.61	[0.25, 0.97]
29060/494	Panic	21.9	10.0	1.08	[0.84, 1.31]	115	20.1	8.0	0.88	[0.67, 1.09]	124	0.22	[−0.04, 0.47]
29060/495	Panic	20.5	9.4	1.03	[0.79, 1.26]	112	20.4	6.6	0.74	[0.54, 0.94]	129	0.31	[0.06, 0.57]
29060/497	Panic	20.5	9.4	0.95	[0.74, 1.16]	128	20.2	6.8	0.75	[0.55, 0.95]	125	0.28	[0.03, 0.52]
29060/637	GAD	26.0	12.4	1.15	[0.96, 1.34]	181	25.9	11.3	1.04	[0.86, 1.22]	183	0.10	[−0.10, 0.31]
29060/641	GAD	23.5	12.3	1.48	[1.33, 1.63]	385	23.9	9.6	1.02	[0.84, 1.20]	180	0.32	[0.14, 0.49]
29060/642	GAD	23.9	11.8	1.32	[1.11, 1.54]	161	23.6	9.5	1.06	[0.86, 1.25]	163	0.26	[0.04, 0.48]
29060/791	GAD	24.4	11.9	1.60	[1.37, 1.84]	163	24.8	10.7	1.44	[1.22, 1.67]	162	0.17	[−0.05, 0.38]
29060A/856	GAD		10.3	1.33	[1.13, 1.53]	177		9.5	1.23	[1.04, 1.43]	181	0.10	[−0.10, 0.31]

GAD = generalized anxiety disorder. Drug benefit denotes the effect size for paroxetine compared to placebo.

A trend toward heterogeneity was observed for the mean effect size difference between paroxetine and placebo, as demonstrated by the indices of heterogeneity (*Q*(11) = 17.63, *p* = .091, *I* ^2^ = 37.61 [95% CI: 12.25,55.64]). A wider range of effect sizes was observed within each treatment group (Paroxetine: *Q*(11) = 57.27, *p*<.001, *I* ^2^ = 80.79 [95% CI: 74.98,85.25]; Placebo: *Q*(11) = 65.39, *p*<.001, *I* ^2^ = 83.18 [95% CI: 78.29,86.96]). These statistics indicate the necessity for moderator analyses to investigate which trial variables influenced study outcomes. Thus, we conducted moderator analyses with both analytic strategies (i.e., the paroxetine-placebo effect sizes and for paroxetine and placebo groups separately).

### HRSA Moderators

The following potential moderators were analyzed: 1) baseline severity of anxiety; 2) indication (i.e., whether the individuals in the trial were treated for panic disorder or for generalized anxiety disorder); 3) length of trial in weeks; and 4) publication status.

**Figure 2:**
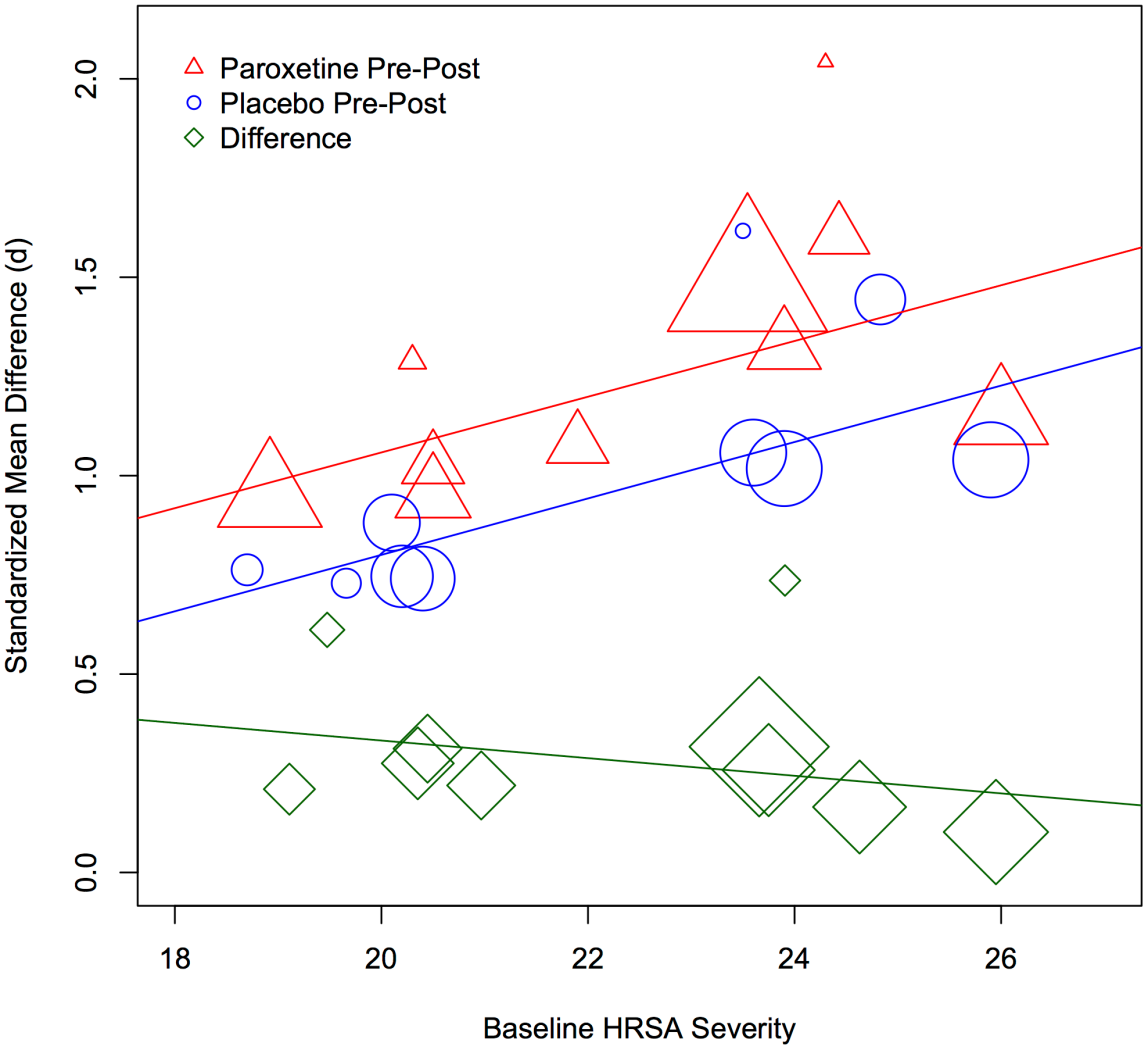
Baseline severity of anxiety and the mean change on the Hamilton Rating Scale for Anxiety (HRSA). The size of the marker reflects the relative weight of the study in the meta-analysis.

There was no significant relationship between baseline anxiety and the paroxetine-placebo effect size difference (*Q*(1) = 1.58, *p* = .208), as shown in Figure 2. A positive relationship was observed between baseline anxiety on the HRSA and effect size for both groups (Paroxetine: *Q*(1) = 21.34, *p*<.001; Placebo: *Q*(1) = 23.51, *p*<.001). These latter effects are consistent with regression to the mean artifact. Baseline severity scores were unavailable for two trials (29060/187 [Panic disorder] and BRL29060A/856 [Generalized anxiety disorder]) that were not included in this analysis.

The effect of indication on treatment response (Table 3) was significant. Panic disorder had a significantly larger drug-placebo difference in terms of the standardized mean difference (*Q*(1) = 5.09, *p* = .024) and the raw change score (*Q*(1) = 6.77, *p* = .009). Mean standardized difference was *d* = 0.36 (95% CI: 0.25,0.46) for panic disorder and *d* = 0.20 (95% CI: 0.11,0.29) for generalized anxiety disorder. Raw score differences were 3.24 points (95% CI: 2.32,4.15) for panic disorder and 1.64 points (95% CI: 0.86,2.42) for generalized anxiety disorder. The effect of indication was also significant within each group (Paroxetine: *Q*(1) = 24.27, *p*<.001; Placebo: *Q*(1) = 32.97, *p*<.001). However, the effects were opposite, with generalized anxiety disorder having higher effect sizes and raw change scores on the HRSA for both groups (Paroxetine: *d* = 1.38 [95% CI: 1.29,1.46] and raw change = 11.79 [95% CI: 11.29,12.30] points; Placebo: *d* = 1.14 [95% CI: 1.05,1.22] and raw change = 10.07 [95% CI: 9.49,10.64] points) compared to panic disorder (Paroxetine: *d* = 1.07 [95% CI: 0.98,1.16] and raw change = 10.06 [95% CI: 9.43,10.68] points; Placebo: *d* = 0.78 [95% CI: 0.69,0.86] and raw change = 7.17 [95% CI: 6.53,7.81] points).

**Table 3:** Moderator effects in trials examining change on the Hamilton Rating Scale for Anxiety.

Indication		Effect Size	95% CI	*Q*(1)	*p*
Paroxetine - Placebo	Panic	0.36	[0.25, 0.46]	5.09	.*024*
	GAD	0.20	[0.11, 0.29]		
Paroxetine	Panic	1.07	[0.98, 1.16]	24.27	*<.001*
	GAD	1.38	[1.29, 1.46]		
Placebo	Panic	0.78	[0.69, 0.86]	32.97	*<.001*
	GAD	1.14	[1.05, 1.22]		
**Publication Status**		**Effect Size**	**95% CI**	***Q*** **(1)**	***p***
Paroxetine - Placebo	Published	0.32	[0.23, 0.40]	3.90	.*048*
	Unpublished	0.17	[0.06, 0.29]		
Paroxetine	Published	1.19	[1.12, 1.27]	3.52	.061
	Unpublished	1.32	[1.21, 1.44]		
Placebo	Published	0.86	[0.79, 0.94]	18.63	<.*001*
	Unpublished	1.15	[1.04, 1.25]		

GAD = generalized anxiety disorder.

Longer trial lengths were significantly associated with larger paroxetine-placebo effect sizes (Intercept = −0.40, Slope = 0.073 [95% CI: 0.023,0.124], *Q*(1) = 8.02, *p* = .005). Within each treatment group, trial length was *inversely* associated with improvement in both groups, and appeared to have a stronger relationship in the placebo group (Paroxetine: Intercept = 2.01, Slope = −0.086 [95% CI: −0.133,−0.038] *Q*(1) = 12.51, *p*<.001; Placebo: Intercept = 2.04, Slope = −0.116 [95% CI: −0.161,−0.072], *Q*(1) = 26.54, *p*<.001). However, this finding is difficult to interpret because it is confounded by differences in study indication. All five trials examining generalized anxiety disorder had a length of eight weeks, and the seven trials examining panic disorder were between 10 and 12 weeks. As described in the previous paragraph, the overall change was larger in both groups for generalized anxiety disorder, which could account for the negative slope within each group, and the drug-placebo difference was larger for panic disorder, which could account for the positive slope in the difference score. Thus, we are unable to make any firm conclusions in this analysis regarding the effect of trial length on anxiolytic response.

There was a significant effect of publication status (Table 3), with larger drug benefits in published trials (*Q*(1) = 3.90, *p* = .048). Published trials (*k* = 8) had a mean drug-placebo effect size of *d* = 0.32 (95% CI: 0.23–0.40), and unpublished trials (*k* = 4) had a mean effect size of *d* = 0.17 (95% CI: 0.06–0.29). This difference appeared to be due to a substantially smaller placebo pre-post effect sizes in published trials (Published: *d* = 0.86 [95% CI: 0.79,0.94], Unpublished: *d* = 1.15 [95% CI: 1.04,1.25], *Q*(1) = 18.63, *p* = .001). The mean pre-post effect size for the paroxetine group was actually marginally smaller for published compared to unpublished trials (Published: *d* = 1.19 [95% CI: 1.12,1.27], Unpublished: *d = *1.32 [95% CI: 1.21,1.44], *Q*(1) = 3.52, *p* = .061).

### Mean Change on the HRSD

Table 4 displays mean baseline severity, mean change, and the standardized mean difference (*d*) for each of the 27 trials reporting change on the HRSD. Baseline severity scores on the HRSD ranged from 19.0 to 30.5 points, all in the ranges of severe to very severe depression [41]. The weighted mean difference between paroxetine and placebo groups across all studies was 2.51 (95% CI: 2.06,2.96) points on the HRSD. The weighted mean effect size difference between the two groups was 0.32 (95% CI: 0.26,0.38). The weighted mean change on the HRSD was 11.00 (95% CI: 10.74,11.26) points for paroxetine and 8.37 (95% CI: 8.02,8.72) points for placebo. The mean pre-post effect size was 1.39 (95% CI: 1.34,1.44) for paroxetine, which was significantly greater (*Q*(1) = 87.62, *p*<.001) than the effect size for placebo (*d* = 1.03 [95% CI: 0.97,1.08]). The magnitude of change in the placebo group was equivalent to 76% of the paroxetine change scores on the HRSD and 74% of the standardized mean difference.

**Table 4:** Baseline scores, mean change, standardized mean change, and sample sizes for all studies reporting change on the Hamilton Rating Scale for Depression.

Protocol	Approval	Paroxetine					Placebo					Drug Benefit	
		Baseline	Change	*d*	95% CI	*n*	Baseline	Change	*d*	95% CI	*n*	*d*	95% CI
29060/01/001	Pre	28.0	13.5	1.66	[0.99, 2.33]	24	27.4	10.5	1.30	[0.71, 1.89]	24	0.37	[−0.20, 0.94]
29060/02/001	Pre	26.6	12.3	1.27	[0.89, 1.65]	51	25.9	6.8	0.70	[0.40, 1.01]	53	0.57	[0.18, 0.97]
29060/02/002	Pre	25.0	10.9	1.24	[0.78, 1.69]	36	24.9	5.8	0.65	[0.27, 1.04]	34	0.60	[0.12, 1.08]
29060/02/003	Pre	28.6	9.7	0.93	[0.50, 1.35]	33	28.9	7.2	0.69	[0.29, 1.08]	33	0.24	[−0.24, 0.73]
29060/02/004	Pre	28.9	12.7	1.87	[1.29, 2.44]	36	27.3	7.6	1.12	[0.70, 1.54]	38	0.76	[0.29, 1.24]
29060/03/001	Pre	24.9	10.8	1.60	[1.11, 2.09]	40	24.8	4.7	0.69	[0.33, 1.06]	38	0.92	[0.45, 1.39]
29060/03/002	Pre	24.9	8.0	1.14	[0.72, 1.55]	40	25.6	6.2	0.88	[0.51, 1.26]	40	0.26	[−0.18, 0.70]
29060/03/003	Pre	25.7	9.9	1.18	[0.76, 1.59]	41	27.0	10.0	1.19	[0.78, 1.60]	42	−0.01	[−0.44, 0.42]
29060/03/004	Pre	27.6	10.4	1.32	[0.86, 1.78]	37	27.0	6.7	0.85	[0.46, 1.24]	37	0.48	[0.02, 0.95]
29060/03/005	Pre	26.1	10.0	1.00	[0.60, 1.39]	40	26.8	4.1	0.41	[0.08, 0.73]	42	0.60	[0.16, 1.05]
29060/03/006	Pre	29.7	9.1	1.10	[0.69, 1.52]	39	28.7	3.0	0.36	[0.02, 0.71]	37	0.76	[0.29, 1.23]
PAR 09	Pre	25.2	9.1	1.28	[1.15, 1.41]	403	24.5	8.2	1.14	[0.78, 1.51]	51	0.12	[−0.17, 0.41]
UK 06	Pre	23.7	6.0	0.97	[0.38, 1.57]	19	24.2	6.2	0.83	[0.31, 1.35]	22	−0.03	[−0.64, 0.58]
UK 12	Pre	22.8	9.1	1.23	[0.57, 1.88]	19	22.3	6.7	0.86	[0.00, 1.73]	10	0.34	[−0.43, 1.11]
UK 09	Pre	26.8	8.8	0.80	[0.26, 1.35]	20	25.5	4.5	0.49	[0.01, 0.97]	21	0.45	[−0.17, 1.07]
PAR 07	Pre	30.5	13.1	1.20	[0.37, 2.03]	13	28.3	10.9	0.99	[0.19, 1.79]	12	0.22	[−0.57, 1.00]
29060/115	Post		10.6	1.23	[1.07, 1.39]	272		9.1	1.09	[0.85, 1.33]	113	0.18	[−0.04, 0.40]
29060/128	Post	25.6	11.8	1.34	[1.19, 1.48]	350	25.7	9.1	1.05	[0.84, 1.26]	136	0.31	[0.11, 0.51]
29060/251	Post	24.5	10.2	1.33	[1.08, 1.57]	120	24.4	8.3	1.07	[0.85, 1.30]	123	0.26	[0.01, 0.51]
29060/327	Post		7.3	1.14	[0.85, 1.42]	79		4.8	0.75	[0.51, 1.00]	83	0.40	[0.09, 0.71]
29060/329	Post	19.0	10.7	1.39	[1.09, 1.68]	90	19.0	9.1	1.16	[0.89, 1.44]	87	0.21	[−0.08, 0.51]
29060/448	Post	23.2	11.9	1.45	[1.25, 1.65]	206	23.4	9.9	1.22	[0.96, 1.48]	101	0.25	[0.01, 0.48]
29060/449	Post	23.8	12.7	1.54	[1.35, 1.74]	218	23.5	10.2	1.24	[0.99, 1.49]	110	0.30	[0.07, 0.53]
29060/487	Post	22.2	12.2	1.68	[1.46, 1.89]	206	22.1	9.5	1.28	[1.02, 1.54]	107	0.37	[0.14, 0.61]
29060/810	Post	23.3	12.0	1.68	[1.50, 1.86]	294	23.8	10.0	1.37	[1.14, 1.60]	142	0.28	[0.08, 0.49]
29060/874	Post	22.5	11.4	1.42	[1.27, 1.57]	334	22.4	8.9	1.10	[0.91, 1.28]	178	0.32	[0.14, 0.50]
112810	Post	22.7	12.7	1.69	[1.50, 1.89]	241	22.6	10.4	1.28	[1.07, 1.48]	171	0.30	[0.10, 0.49]

“Approval” column designates whether the study was submitted as part of the original approval submission to the FDA or whether it was conducted following FDA approval in 1991 or later. Drug benefit denotes the effect size for paroxetine compared to placebo.

Indices of heterogeneity did not indicate statistically significant heterogeneity in the effect size difference between paroxetine and placebo across trials (*Q*(26) = 26.54, *p* = .434, *I* ^2^ = 2.04 [95% CI: −29.25,25.75]), although we did detect significant heterogeneity within each group (Paroxetine: *Q*(26) = 63.21, *p*<.001, *I* ^2^ = 58.87 [95% CI: 49.14–66.73]; Placebo: *Q*(26) = 80.63, *p*<.001, *I* ^2^ = 67.75 [95% CI: 60.63,73.59]). Nevertheless, as planned when designing the study, moderator analyses were conducted for both types of analyses.

### HRSD Moderators

We analyzed the following moderators to determine whether the variables could account for variance in effect size across trials: 1) baseline severity of depression; 2) approval status; 3) length of treatment in weeks; and 4) publication status.

**Figure 3:**
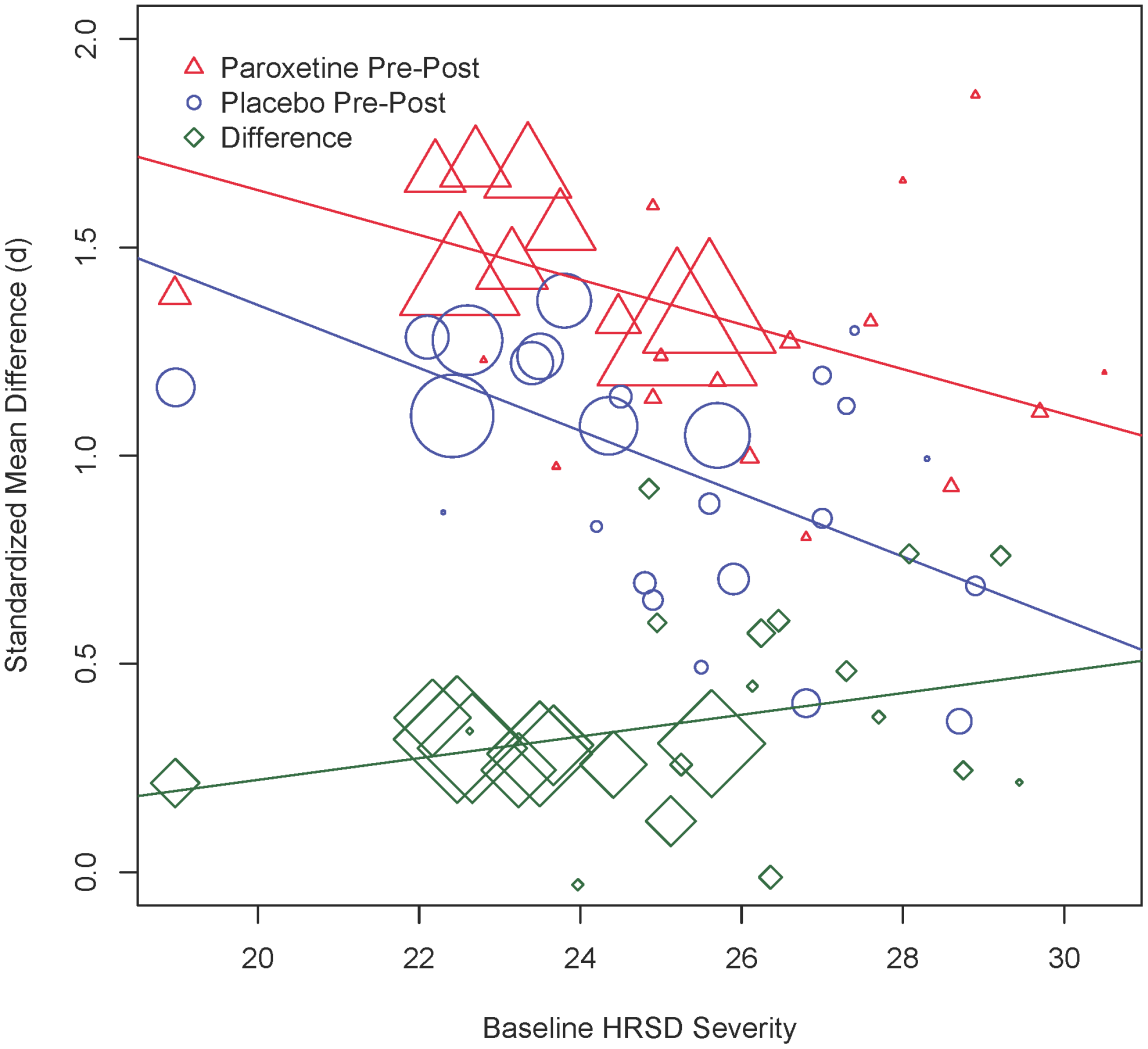
Baseline severity of depression and the mean change on the Hamilton Rating Scale for Depression (HRSD). The size of the marker reflects the relative weight of the study in the meta-analysis.

Figure 3 displays the relationship between baseline severity of depression on the HRSD and treatment outcome. The benefit of paroxetine over placebo was not significantly related to baseline severity (*Q*(1) = 3.01, *p* = .083), although there was a trend towards a greater benefit at higher baseline severities. The predicted paroxetine-placebo effect size at a baseline severity of HRSD = 19 was *d = *0.20 (95% CI: 0.03,0.36) and *d* = 0.48 (95% CI: 0.29,0.68) at a baseline severity of HRSD = 30. Greater baseline severity of depression was associated with smaller pre-post effect sizes in both paroxetine (*Q*(1) = 15.45, *p*<.001) and placebo (*Q*(1) = 28.23, *p*<.001) groups. These effects are opposite from those expected based on regression to the mean artifact.

A comparison of the trials submitted for the original FDA approval (pre-approval, *k* = 16) versus trials conducted after approval (post-approval, *k* = 11), shown in Table 5, revealed that the mean paroxetine-placebo effect size did not differ significantly as a function of approval status (*Q*(1) = 3.27, *p* = .077), although there was a trend towards a greater drug-placebo benefit in pre-approval trials (Pre-Approval: *d* = 0.41 [95% CI: 0.30,0.53]; Post-Approval: *d = *0.29 [95% CI: 0.22,0.36]). However, we observed a significant effect within both groups, with larger mean standardized differences in the post-approval trials (Table 5). For paroxetine, the mean effect size for pre-approval trials was *d* = 1.24 (95% CI: 1.15,1.33), compared to *d* = 1.45 (95% CI: 1.39,1.50) for post-approval (*Q*(1) = 14.43, *p*<.001). For placebo, the mean effect sizes were *d* = 0.77 (95% CI: 0.67,0.87) and *d* = 1.14 (95% CI: 1.07,1.21) for the pre- and post-approval trials, respectively (*Q*(1) = 35.01, *p*<.001).

**Table 5:** Moderator effects in trials examining change on the Hamilton Rating Scale for Depression.

Pre- vs. Post-Approval		Effect Size	95% CI	*Q*(1)	*p*
Paroxetine - Placebo	Pre-Approval	0.41	[0.30, 0.53]	3.27	.071
	Post-Approval	0.29	[0.22, 0.36]		
Paroxetine	Pre-Approval	1.24	[1.15, 1.33]	14.43	*<.001*
	Post-Approval	1.45	[1.39, 1.50]		
Placebo	Pre-Approval	0.77	[0.67, 0.87]	35.01	*<.001*
	Post-Approval	1.14	[1.07, 1.21]		
**Publication Status**		**Effect Size**	**95% CI**	***Q*** **(1)**	***p***
Paroxetine - Placebo	Published	0.36	[0.27, 0.44]	1.50	.221
	Unpublished	0.28	[0.20, 0.37]		
Paroxetine	Published	1.41	[1.35, 1.48]	1.45	.229
	Unpublished	1.35	[1.28, 1.43]		
Placebo	Published	0.99	[0.91, 1.07]	1.46	.227
	Unpublished	1.06	[0.98, 1.15]		

Pre-approval and Post-approval refer to whether the trial was included as part of the original approval submission to the FDA (*k* = 16) or whether it was conducted following FDA approval in 1991 or later (*k* = 11).

**Figure 4:**
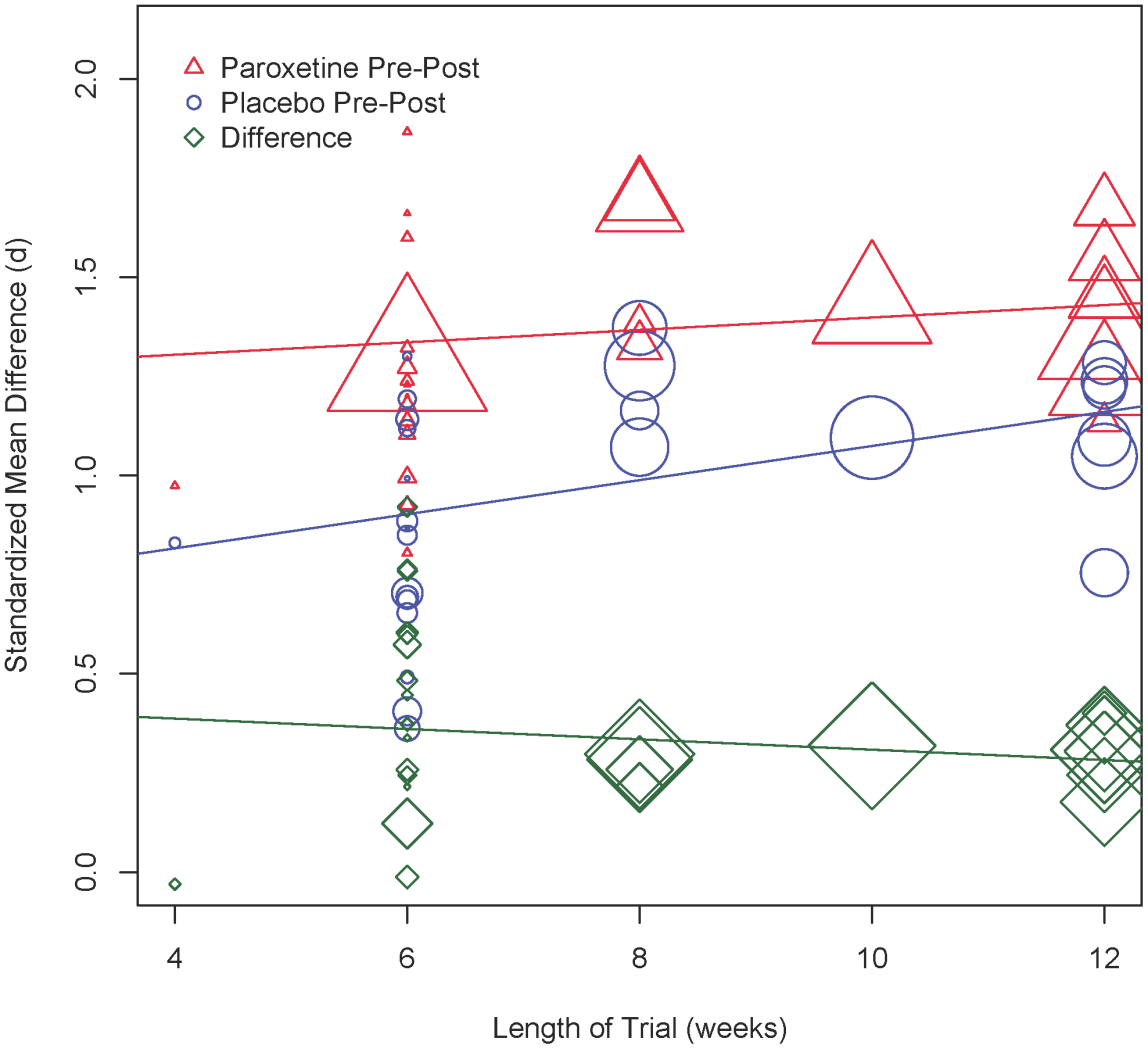
Trial duration (in weeks) and the mean change on the Hamilton Rating Scale for Depression (HRSD). The size of the marker reflects the relative weight of the study in the meta-analysis.

An examination of the effect of trial duration on efficacy (Figure 4) revealed that the benefit of paroxetine over placebo was not significantly associated with trial duration (*Q*(1) = 1.30, *p* = .254). Likewise, the response to paroxetine did not significantly differ as a function of study length (*Q*(1) = 2.62, *p* = .105), although the mean change in the placebo group was significantly larger in longer studies (*Q*(1) = 13.74, *p*<.001).

The weighted mean difference between paroxetine and placebo was not significantly different between published and unpublished trials (Table 5; *Q*(1) = 1.50, *p* = .221). Published trials (*k* = 16) had a weighted mean effect size of *d* = 0.36 (95% CI: 0.27,0.44) and unpublished trials (*k* = 11) had an effect size of *d* = 0.28 (95% CI: 0.20,0.37).

### Comparison of Change on the HRSA and HRSD

A comparison of the standardized mean difference between the change on the two scales indicated that the paroxetine-placebo effect size did not significantly differ between the HRSA and the HRSD (HRSA: *d* = 0.27 [95% CI: 0.20,0.33], HRSD: *d* = 0.32 [95% CI: 0.26,0.38], *Q*(1) = 1.41, *p* = .235). The mean pre-post effect size for paroxetine treatment was significantly larger (*Q*(1) = 14.55, *p*<.001) for the HRSD (*d* = 1.39 [95% CI: 1.34,1.44], *k* = 27) than for the HRSA (*d* = 1.23 [95% CI: 1.17,1.30], *k* = 12). A non-significant trend (*Q*(1) = 2.38, *p* = .123) was observed in the placebo group for larger pre-post effect sizes on the HRSD (*d* = 1.03 [95% CI: 0.97,1.08]) than on the HRSA (*d* = 0.96 [95% CI: 0.90,1.02]).

## Discussion

The current analysis is the first evaluation of the efficacy of an SSRI medication in the treatment of multiple anxiety disorders, and the first to utilize a complete database of published and unpublished trials sponsored by the drug’s manufacturer. Our results indicated that paroxetine presented a modest benefit over placebo in the treatment of anxiety and depression, with mean change score differences of 2.3 and 2.5 points on the HRSA and HRSD, respectively. The standardized mean difference of paroxetine over placebo was *d* = 0.27 and *d* = 0.32 for the treatment of anxiety and depression, respectively. Put another way, the average symptom reduction for an individual treated with paroxetine fell at the 61^st^ percentile for individuals who received placebo for anxiety, and at the 63^rd^ percentile for individuals who received placebo for depression. The difference of *d* = 0.32 in the treatment of depression is consistent with previous meta-analyses of antidepressant efficacy [5], [7]. The mean treatment response did not significantly differ between treatment of anxiety and treatment of depression. We demonstrated that individuals given placebo exhibited 79% of the magnitude of change compared to paroxetine. We also provided further support for the large magnitude of the changes in placebo groups in the treatment of depression (76% compared to paroxetine).

Several moderator variables were significantly associated with pre-post effect sizes for paroxetine and placebo on both the HRSA and the HRSD. For anxiety, we found that higher baseline severity was unrelated to drug-placebo differences, although higher severity was associated with greater changes in both paroxetine and placebo groups. Efficacy was superior in the treatment of panic disorder compared to generalized anxiety disorder; however, the overall response to both paroxetine and placebo was larger for generalized anxiety disorder. Samples with higher baseline severities were associated with lower changes in both paroxetine and placebo groups in the treatment of depression, an effect that is especially peculiar given that it is opposite to that predicted by regression toward the mean. Longer treatment was associated with larger pre-post placebo effect sizes in the treatment of depression. The increase in the symptom reduction in the placebo group in longer trials for the treatment of depression is especially interesting, given the widespread belief that placebo effects are short lived.

The magnitude of change in the placebo group was greater than 75% of the paroxetine response in the treatment of both anxiety and depression. Large effect sizes in placebo groups have been reported in the treatment of other conditions as well. However, these changes compared to the drug effect sizes do not appear to be as large as those observed in antidepressant trials in the treatment of depression and anxiety. For example, a review of the placebo effect compared to active medications (including antidepressants and anticonvulsants) in the treatment of pain associated with fibromyalgia revealed that the mean change in placebo groups accounted for 45% of the drug response [44]. This same review found that pain reduction in the placebo groups compared to the drug response in individuals with painful peripheral diabetic neuropathy was 62% [44]. Similar meta-analytic reviews have found that mean change in placebo groups replicates about 40% of drug responses in global symptom reduction during treatment of irritable bowel syndrome [45], [46]. In a meta-analysis of change in placebo compared to drug groups in the treatment of symptoms of chronic fatigue syndrome, the mean placebo effect replicated only 20% of the drug response [47]. Thus, the replication of greater than 75% of the drug response indicates that the magnitude of the placebo effect is especially large in the treatment of anxiety and depression.

Given the similar efficacy between paroxetine and other second-generation antidepressants in the treatment of depression [7], [12], [48], it is possible that a similar magnitude of placebo effect sizes are present in the treatment of anxiety disorders with other antidepressants. However, further research will be necessary to support this proposition. The current analysis indicates that the published literature represents an overestimate of the true efficacy of paroxetine in the treatment of anxiety.

Although the differences between drug and placebo are statistically significant, whether antidepressants produce clinically significant benefits has been a topic of debate in recent years. However, to date there has been no consensus regarding what constitutes a clinically significant benefit. In their 2004 guidelines for the treatment of depression, the National Institute of Health and Clinical Excellence (NICE) proposed a mean drug-placebo standardized mean difference (SMD) ≥0.50 or a difference of at least three points on the HRSD as criteria for clinical significance [43]. Based on these criteria, the mean antidepressant benefit in a previous meta-analysis of trials submitted to the FDA [7] was clinically significant only in the most severe cases of depression (baseline HRSD ≥28). In a subsequent revision of their guidelines for the treatment of depression [42], NICE replaced the term “clinical significance” with “clinical importance.” Although they did not specify their criteria for determining whether an effect was clinically important, their comparisons of SSRI-placebo differences in HRSD scores were the same as in the earlier guidelines, and the same conclusions regarding “clinical importance” were reached as had been reached with respect to “clinical significance” in 2004. Specifically, the overall difference between SSRIs and placebo (SMD = 0.34) was described as “unlikely to be of clinical importance” (pg. 317). According to these criteria, the mean difference between paroxetine and placebo in the current analyses fell short of clinical significance for the treatment of both anxiety and depression.

The NICE criteria have been criticized for being arbitrary and lacking empirical justification [49]. However, a recent analysis of raw data from 43 antidepressant trials [50] compared HRSD change scores with clinician ratings of improvement on the Clinical Global Impressions Scale (CGI) [51] to establish the clinical relevance of HRSD scores. They found that change of three points or less on the HRSD corresponded to a clinician rating of “No Change” on the CGI. That is, changes of three points or less did not correspond to a clinically detectable change according to this clinician-rated measure. Thus, the drug-placebo differences that have been observed in the current and previous antidepressant meta-analyses [7], [28], while statistically significant, appear to be of marginal clinical significance.

These findings have important clinical implications. The obvious alternative for the treatment of both anxiety and depression is psychotherapy intervention. However, direct comparisons of acute phase treatment for pharmacotherapy and psychotherapy in the treatment of major depression generally have yielded no significant differences between the treatment modalities [52]–[54]. Fewer clinical trials have directly compared antidepressants and psychotherapy in the treatment of anxiety disorders, although the available literature indicates similar comparability between antidepressants and psychotherapy. For example, one study [55] found that that acute phase cognitive-behavioral therapy yields comparable efficacy to imipramine in the treatment of panic disorder. Another study [56] found comparable 12-week efficacy between sertraline and cognitive-behavioral therapy in the treatment of childhood anxiety disorders. Overall, antidepressants, psychotherapy, and placebo all yield substantial changes in symptomatology, and are superior to no-treatment control groups [9]. Thus, in terms of treatment, the specific type of intervention may be less important than simply getting patients involved in some sort of active therapy program [53].

When given two seemingly equivalent alternatives with regard to symptom reduction, the decision may come down to patient preference and to the safety profile associated with the treatment. A meta-analysis of patient preferences when given the choice between psychological and pharmacologic treatment [57] revealed that 75% of patients prefer psychological intervention across 30 studies comprising individuals seeking treatment for depression or anxiety disorders. Paroxetine and other SSRIs have also been associated with a number of adverse events during treatment. Greater than 70% of patients report treatment-emergent symptoms of sexual dysfunction including reduced desire, arousal, and/or orgasm dysfunction, compared to less than 10% of individuals who received placebo [58]. Other adverse reported effects include drowsiness and weight gain, observed in greater than 7% of patients taking SSRIs [59]. Infrequent but severe symptoms such as serotonin syndrome [60] and increased suicidal ideation in younger adults [61], [62] have also been reported. Additionally, abrupt withdrawal can result in a discontinuation syndrome in 66% of patients taking paroxetine, including symptoms of dizziness, worsened mood, agitation, headache, and nausea [63]. It is also notable that the frequency of adverse events many be underestimated in the clinical literature, as patients with depression are far more likely to self-report side effects on questionnaires than report them to physicians as is typical during clinical trials [64].

Although meta-analyses have indicated comparable efficacy between antidepressants and psychotherapy during acute stage treatment, their comparability is not as clear for long-term treatment. One study [54] found that individuals who had received “bona fide” psychotherapy from trained professionals displayed greater symptom reduction compared to those who had received SSRI treatment at post-acute phase follow-up ranging from 18 to 40 weeks (*d* = 0.29, *k* = 6). Another meta-analysis [52] of long-term naturalistic follow-up between individuals who were randomized to either acute-phase pharmacotherapy or psychotherapy in the treatment of depression across 11 studies revealed an advantage for psychotherapy at an average follow-up length of 15 months. Moreover, length of follow-up was a significant moderator such that the advantage of psychotherapy over medication was superior at longer follow-up intervals. The authors suggest that psychotherapy offers a “prophylactic effect” resulting in its long-term superiority over medications [52]. In an additional analysis of nine studies, Imel et al. [52] demonstrated that acute-phase discontinued psychotherapy was as efficacious as continued pharmacotherapy at an average follow-up interval of 14 months. That is, short-term psychotherapy (between 7 and 24 sessions) provided an equivalent long-term benefit to continuous medication usage. These findings can help to explain why antidepressants are frequently used for chronic treatment; more than 60% of individuals who take antidepressants have done so for longer than 2 years, and greater than 30% use them for 5 years or more [65]. In sum, the drawbacks to antidepressant usage and their modest benefit compared to placebo should be seriously considered before they are chosen as the primary treatment for depression or anxiety.

A limitation of the current work is that the trial database was limited to studies sponsored by GlaxoSmithKline, and does not include any additional trials that may have been conducted by independent researchers. Additionally, it is possible that GlaxoSmithKline omitted some of the outcome indices from the trial summaries posted online. A further limitation of the current analysis is that baseline severity and change were evaluated with the mean values for each group. An analysis including baseline values and response at the individual patient level would afford more power in determining a more precise estimate for the relative benefit of paroxetine over placebo at differing levels of baseline severity. The standard result summaries provided by the GlaxoSmithKline Clinical Trial Register provide baseline values and change scores only at the group level. These result summary documents also provided limited information regarding the ways in which the trials were conducted, which hindered our ability to conduct a thorough analysis for study quality. However, it appears that clinical trial sponsors are recognizing the importance of the availability of patient-level data. Several sponsors, including GlaxoSmithKline, have committed to posting patient-level study results online at Clinical Study Data Request [66]. According to this site, GlaxoSmithKline plans to have data for all studies conducted after December 2000 freely available some time in 2015, with further studies available upon request. This site may be a valuable resource for future meta-analyses of drug efficacy.

A recent study conducted a patient-level analysis examining the relationship between baseline severity and antidepressant efficacy in the treatment of depression [39]. This study analyzed individuals from six double-blind, placebo-controlled studies of paroxetine and imipramine and found that the drug-placebo difference was greater than three points on the HRSD only at baseline severity levels of 25 and above. In fact, for individuals with mild or moderate depression (HRSD ≤18), the drug benefit was less than one point on the HRSD. This finding is concerning given that among Americans aged 12 years or older, approximately 19% and 28% of individuals with mild and moderate depression, respectively, take antidepressants [65].

In conclusion, paroxetine provides only a modest benefit over placebo in treating symptoms of anxiety based on the available evidence. In addition, the current study supports previous work [7] indicating that paroxetine treatment presents only a modest benefit over placebo in the treatment of depression.

## Supporting Information

Checklist S1 PRISMA checklist.
(DOC)

Figure S1 Baseline severity of anxiety and the mean change on the Hamilton Rating Scale for Anxiety (HRSA). The size of the marker reflects the relative weight of the study in the meta-analysis. Random effects assumptions were used in the analyses. The relationship between baseline severity and effect size was marginally significant for paroxetine (*p* = .069) and statistically significant for placebo (*p* = .020), but not for the difference between paroxetine over placebo (*p* = .401).
(TIFF)

Figure S2 Baseline severity of depression and the mean change on the Hamilton Rating Scale for Depression (HRSD). The size of the marker reflects the relative weight of the study in the meta-analysis. Random effects assumptions were used in the analyses. The relationship between baseline severity and effect size was statistically significant for paroxetine (*p* = .029) and for placebo (*p* = .004), but not for the difference between paroxetine over placebo (*p* = .094).
(TIFF)

Figure S3 Trial duration (in weeks) and the mean change on the Hamilton Rating Scale for Depression (HRSD). The size of the marker reflects the relative weight of the study in the meta-analysis. Random effects assumptions were used in the analyses. The relationship between trial length and effect size was not statistically significant for paroxetine (*p* = .126), but was statistically significant for placebo (*p* = .017). The relationship was not statistically significant for the difference between paroxetine over placebo (*p* = .297).
(TIFF)

Results S1 Contains Table S1 and Table S2.
(DOCX)
