# Supplementary figures and images for: The Efficacy of Paroxetine and Placebo in Treating Anxiety and Depression: A Meta-Analysis of Change on the Hamilton Rating Scales
Source: PLoS One. 2014 Aug 27;9(8):e106337. doi: 10.1371/journal.pone.0106337 (PMC4146610; doi:10.1371/journal.pone.0106337)

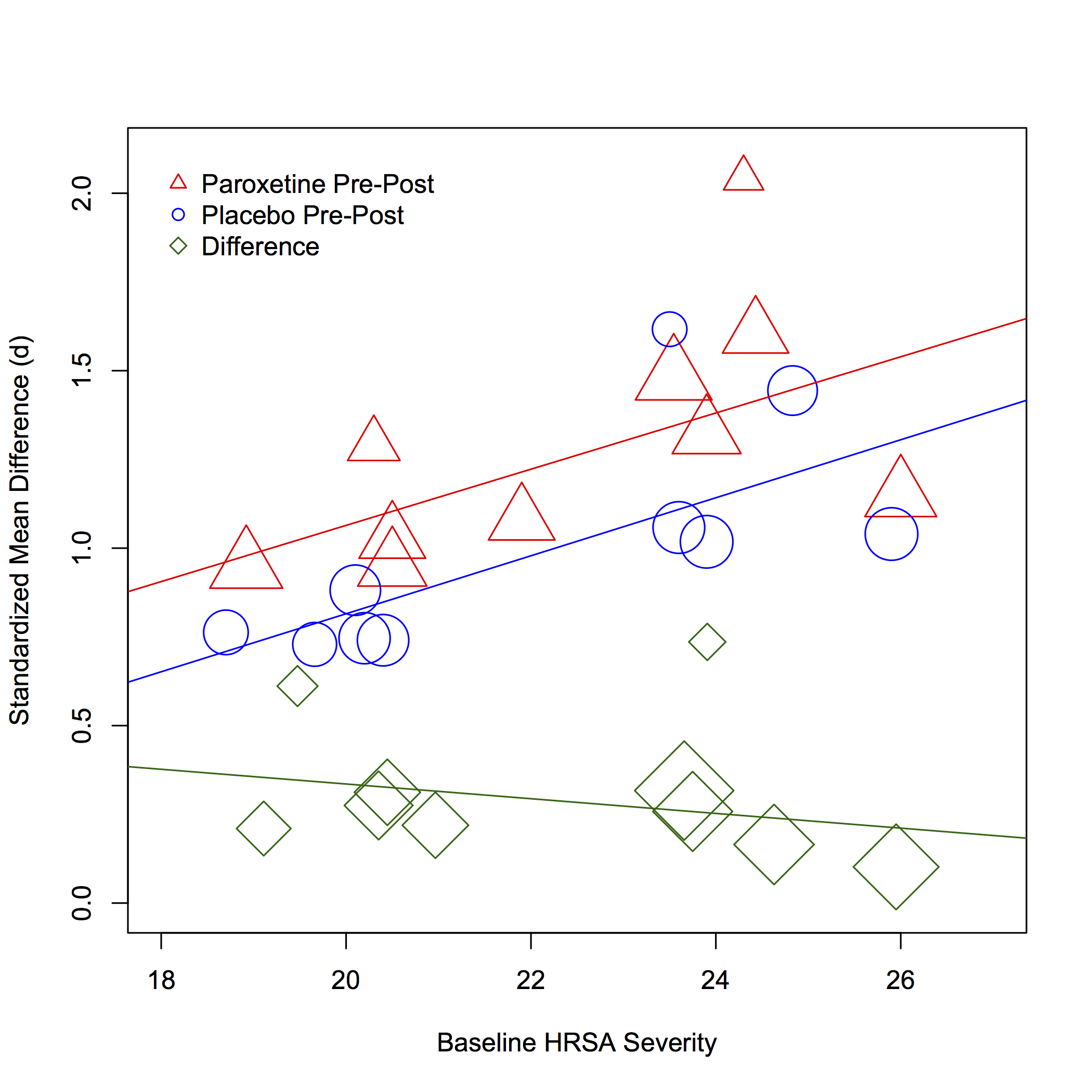

Supplement: Figure S1 — Baseline severity of anxiety and the mean change on the Hamilton Rating Scale for Anxiety (HRSA). The size of the marker reflects the relative weight of the study in the meta-analysis. Random effects assumptions were used in the analyses. The relationship between baseline severity and effect size was marginally significant for paroxetine (p = .069) and statistically significant for placebo (p = .020), but not for the difference between paroxetine over placebo (p = .401). (TIFF) [file pone.0106337.s002.tiff]

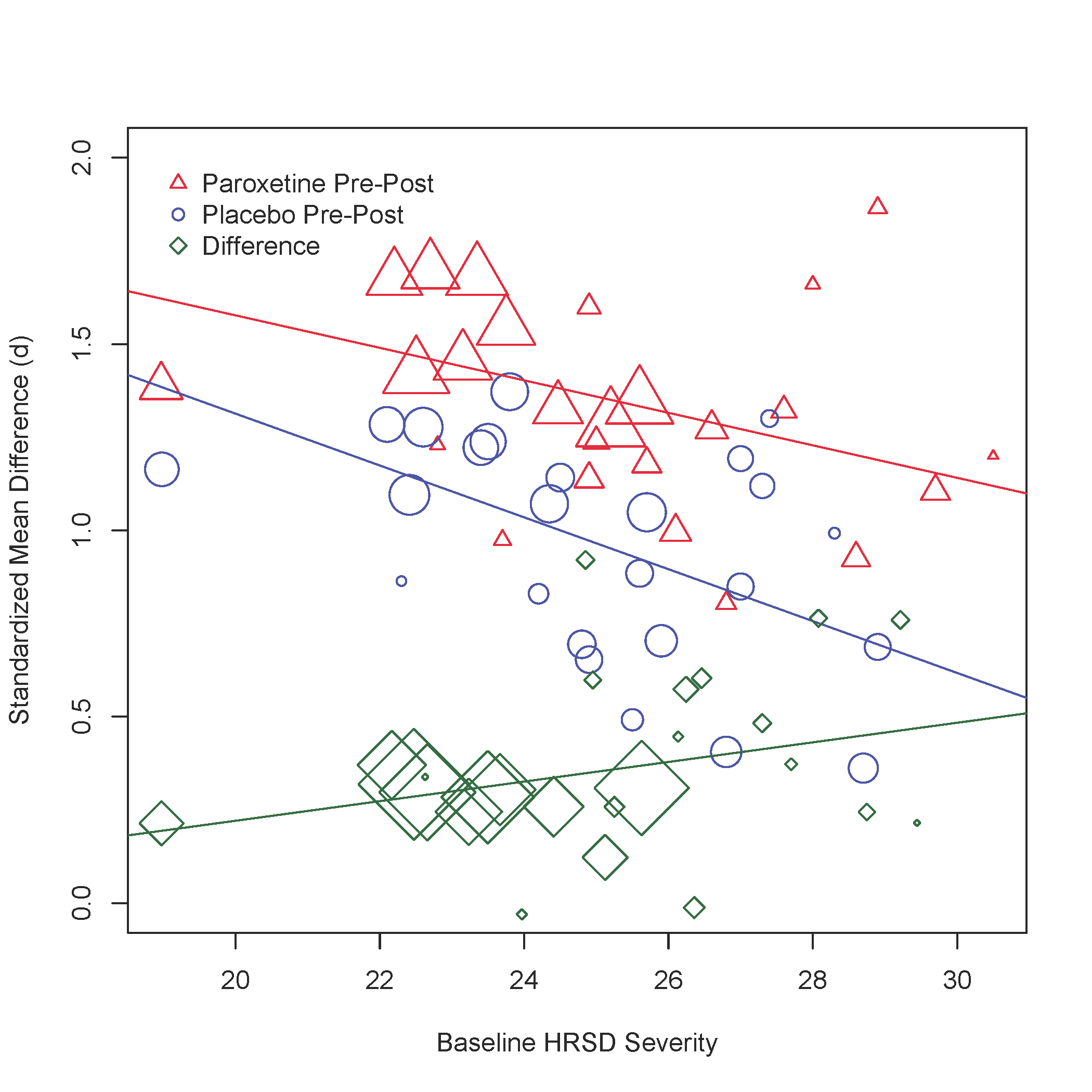

Supplement: Figure S2 — Baseline severity of depression and the mean change on the Hamilton Rating Scale for Depression (HRSD). The size of the marker reflects the relative weight of the study in the meta-analysis. Random effects assumptions were used in the analyses. The relationship between baseline severity and effect size was statistically significant for paroxetine (p = .029) and for placebo (p = .004), but not for the difference between paroxetine over placebo (p = .094). (TIFF) [file pone.0106337.s003.tiff]

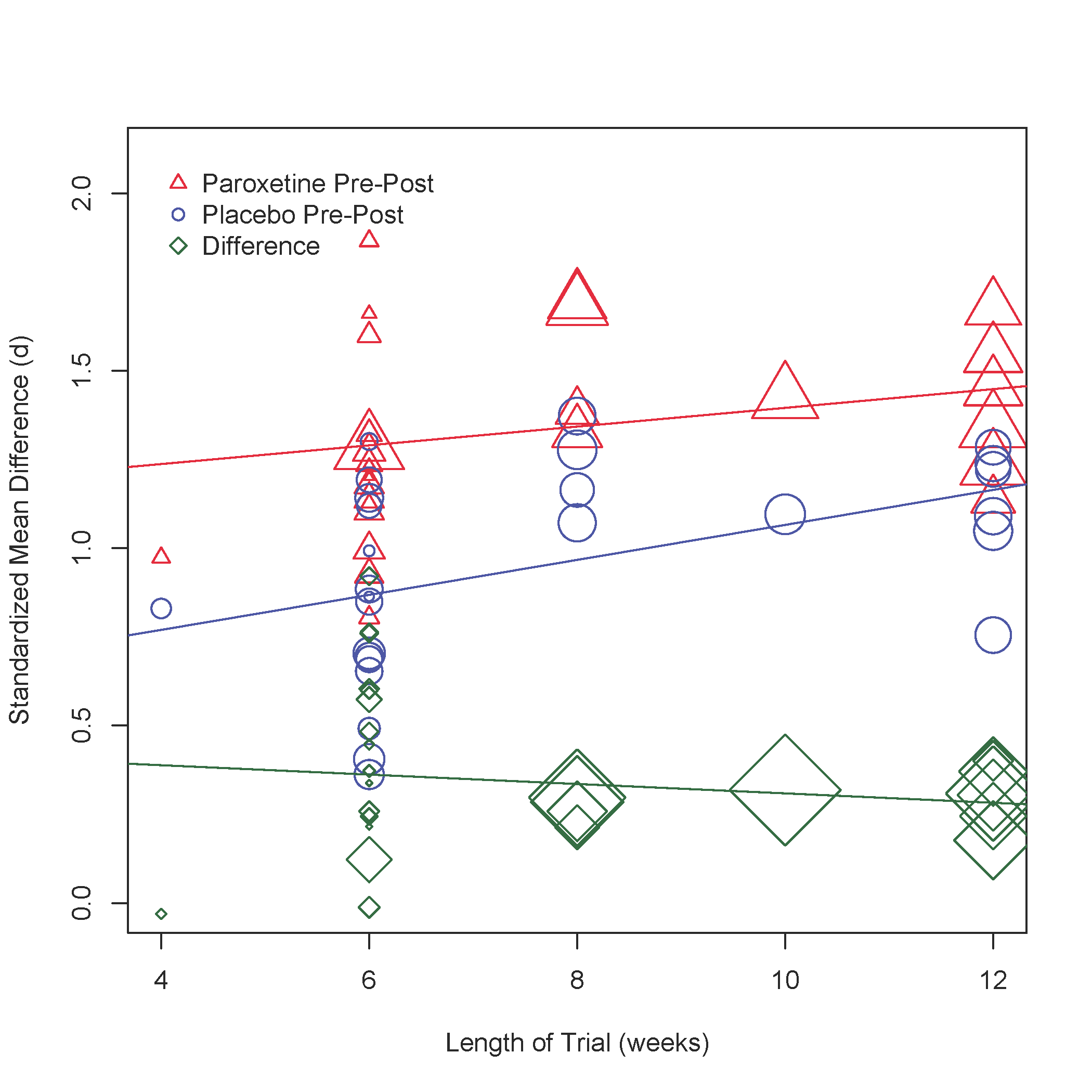

Supplement: Figure S3 — Trial duration (in weeks) and the mean change on the Hamilton Rating Scale for Depression (HRSD). The size of the marker reflects the relative weight of the study in the meta-analysis. Random effects assumptions were used in the analyses. The relationship between trial length and effect size was not statistically significant for paroxetine (p = .126), but was statistically significant for placebo (p = .017). The relationship was not statistically significant for the difference between paroxetine over placebo (p = .297). (TIFF) [file pone.0106337.s004.tiff]
