# Supplementary material for: The Efficacy of Paroxetine and Placebo in Treating Anxiety and Depression: A Meta-Analysis of Change on the Hamilton Rating Scales
Source: PLoS One. 2014 Aug 27;9(8):e106337. doi: 10.1371/journal.pone.0106337 (PMC4146610; doi:10.1371/journal.pone.0106337)
Supplement: Results S1 — Contains Table S1 and Table S2. (DOCX) [file pone.0106337.s005.docx]

**Table S1.** Main effects and moderator effects in trials examining change on the Hamilton Rating Scale for Anxiety (HRSA), using random-effects assumptions. The main effects are presented both as the raw change in points on the HRSA and as the standardized mean difference (*d*). Moderator effects were conducted using the standardized mean difference. GAD = Generalized Anxiety Disorder, MDD = Major Depressive Disorder accompanied by symptoms of anxiety

| **Main Effects** |  | **Value** | **95% CI** |  | ***Z*** | ***p*** |
| --- | --- | --- | --- | --- | --- | --- |
| Paroxetine - Placebo | HRSA Change | 2.41 | [1.67. 3.16] |  | 6.36 | *< .001* |
|  | Std. Mean Diff | 0.28 | [0.19, 0.37] |  | 6.08 | *< .001* |
|  |  |  |  |  |  |  |
| Paroxetine | HRSA Change | 10.94 | [9.98, 11.89] |  | 22.50 | *< .001* |
|  | Std. Mean Diff | 1.25 | [1.10, 1.39] |  | 16.80 | *< .001* |
|  |  |  |  |  |  |  |
| Placebo | HRSA Change | 8.41 | [7.33, 9.49] |  | 15.26 | *< .001* |
|  | Std. Mean Diff | 0.98 | [0.82, 1.13] |  | 12.51 | *< .001* |
| **Indication** |  | **Effect Size** | **95% CI** |  | ***Q*(1)** | ***p*** |
| Paroxetine - Placebo | Panic | 0.37 | [0.24, 0.50] |  | 4.46 | *.035* |
|  | GAD | 0.20 | [0.11, 0.29] |  |  |  |
|  |  |  |  |  |  |  |
| Paroxetine | Panic | 1.14 | [0.96, 1.32] |  | 4.00 | *.045* |
|  | GAD | 1.37 | [1.23, 1.52] |  |  |  |
|  |  |  |  |  |  |  |
| Placebo | Panic | 0.82 | [0.65, 0.99] |  | 8.20 | *.004* |
|  | GAD | 1.15 | [1.00, 1.30] |  |  |  |
| **Publication Status** |  | **Effect Size** | **95% CI** |  | ***Q*(1)** | ***p*** |
| Paroxetine - Placebo | Published | 0.32 | [0.23, 0.40] |  | 1.35 | .246 |
|  | Unpublished | 0.20 | [0.02, 0.38] |  |  |  |
|  |  |  |  |  |  |  |
| Paroxetine | Published | 1.21 | [1.01, 1.40] |  | 0.85 | .355 |
|  | Unpublished | 1.34 | [1.14, 1.54] |  |  |  |
|  |  |  |  |  |  |  |
| Placebo | Published | 0.89 | [0.73, 1.05] |  | 2.46 | .117 |
|  | Unpublished | 1.13 | [0.88, 1.38] |  |  |  |

**Table S2.** Main effects and moderator effects in trials examining change on the Hamilton Rating Scale for Depression (HRSD). Pre-approval and Post-approval refer to whether the trial was included as part of the original approval submission to the FDA (*k* = 16) or whether it was conducted following approval in 1991 or later (*k* = 11). Analyses were conducted using random effects assumptions. The main effects are presented both as the raw change in points on the HRSA and as the standardized mean difference (*d*). Moderator effects were conducted using the standardized mean difference.

| **Main Effects** |  | | **Value** | **95% CI** |  | ***Z*** | ***p*** |
| --- | --- | --- | --- | --- | --- | --- | --- |
| Paroxetine - Placebo | | HRSD Change | 2.53 | [2.05, 3.02] |  | 10.23 | *< .001* |
|  | | Std. Mean Diff | 0.32 | [0.26, 0.38] |  | 10.54 | *< .001* |
|  | |  |  |  |  |  |  |
| Paroxetine | | HRSD Change | 10.73 | [10.06, 11.39] |  | 31.59 | *< .001* |
|  | | Std. Mean Diff | 1.36 | [1.27, 1.45] |  | 30.78 | *< .001* |
|  | |  |  |  |  |  |  |
| Placebo | | HRSD Change | 7.85 | [7.05, 8.65] |  | 19.24 | *< .001* |
|  | | Std. Mean Diff | 0.98 | [0.87, 1.08] |  | 17.80 | *< .001* |
| **Pre- vs. Post-Approval** | | | **Effect Size** | **95% CI** |  | ***Q*(1)** | ***p*** |
| Paroxetine - Placebo | | Pre-Approval | 0.43 | [0.28, 0.57] |  | 2.91 | .088 |
|  | | Post-Approval | 0.29 | [0.22, 0.36] |  |  |  |
|  | |  |  |  |  |  |  |
| Paroxetine | | Pre-Approval | 1.23 | [1.13, 1.33] |  | 8.38 | *.004* |
|  | | Post-Approval | 1.45 | [1.34, 1.56] |  |  |  |
|  | |  |  |  |  |  |  |
| Placebo | | Pre-Approval | 0.79 | [0.65, 0.93] |  | 16.48 | *< .001* |
|  | | Post-Approval | 1.15 | [1.05, 1.24] |  |  |  |
| **Publication Status** | |  | **Effect Size** | **95% CI** |  | ***Q*(1)** | ***p*** |
| Paroxetine - Placebo | | Published | 0.38 | [0.27, 0.48] |  | 1.94 | .163 |
|  | | Unpublished | 0.28 | [0.20, 0.37] |  |  |  |
|  | |  |  |  |  |  |  |
| Paroxetine | | Published | 1.37 | [1.25, 1.50] |  | 0.21 | .645 |
|  | | Unpublished | 1.33 | [1.21,1.46] |  |  |  |
|  | |  |  |  |  |  |  |
| Placebo | | Published | 0.93 | [0.76, 1.09] |  | 1.31 | .252 |
|  | | Unpublished | 1.05 | [0.93, 1.17] |  |  |  |
